# Supplementary material for: Sharing the load: How a personally coloured calculator for grapheme-colour synaesthetes can reduce processing costs
Source: PLoS One. 2021 Sep 22;16(9):e0257713. doi: 10.1371/journal.pone.0257713 (PMC8457480; doi:10.1371/journal.pone.0257713)
Supplement: S1 Fig — (PDF) [file pone.0257713.s001.pdf]

S1 Fig:

Pre-randomise and balanced tests.

No.	Test 1	Test 2	Test 3
1	5 + 8 =	7 + 4 =	6 + 9 =
2	43 + 58 =	62 +74 =	96 + 35 =
3	315 + 759 =	486 + 702 =	695 + 482 =
4	5731 + 4256 =	3980 + 4716 =	8063 + 1468 =
5	42385 + 38709 =	20981 + 59215 =	70092 + 10264 =
6	496230 + 540923 =	741320 + 567390 =	633940 + 547721 =
7	9 – 4 =	6 – 3 =	8 – 4 =
8	86 – 54 =	78 – 49 =	94 – 74 =
9	456 – 857 =	361 – 902 =	574 – 693 =
10	8546 – 2750 =	8435 – 2193 =	6593 – 1827 =
11	21967 – 38460 =	53982 – 72106 =	49607 – 62318 =
12	734912 – 395680 =	691324 – 384605 =	590142 – 287163 =
13	42 x 6.8 =	31 x 8.3 =	75 x 2.8 =
14	421 x 859 =	509 x 673 =	712 x 472 =
15	5.432 x 391 =	6.429 x 327 =	4.278 x 493 =
16	1575 x 1169 =	1665 x 1759 =	1097 x 1659 =
17	71448 ÷ 104 =	66650 ÷ 155 =	77967 ÷ 176 =
18	5828 ÷ 31 =	5576 ÷ 41 =	5678 ÷ 34 =
19	71603 ÷ 104 =	75510 ÷ 86 =	70835 ÷ 121 =
20	50494 ÷ 97 =	76961 ÷ 81 =	45169 ÷ 113 =
21	125.60 - 89.52 x (3.7 + 6.3) =	106.14 - 61.87 x (4.5 + 5.8) =	142.4 - 89.25 x (3.9 + 8.4) =
22	4 + 5 x (1 - 3) =	3 + 5 x (1 - 4) =	3 + 6 x (2 - 4) =
23	69 x 9.2 + 24 - 36.43 =	94 x 6.6 + 53 - 85.23 =	51 x 6.7 + 53 - 78.52 =
24	93 x 1.8 - 125.43 + 74 =	78 x 8.9 - 97.53 + 101 =	87 x 7.4 - 142.96 + 82 =
25	27 - 2.24 ÷ 1.6 + 12 =	43 - 3.61 ÷ 1.9 + 31 =	52 - 1.82 ÷ 1.3 + 14 =
26	24 + 80.96 ÷ (10-1.2) – 5 =	43 + 61.5 ÷ (9-1.5) – 32 =	36 + 36.57 ÷ (8-1.1) – 11 =
27	2079 ÷ 33 - 142 + 52 =	7980 ÷ 84 - 127 + 86 =	2673 ÷ 81 - 74 + 132 =
28	1428 ÷ (30+4) + 24.84 - 43.21 =	2088 ÷ (50+8) + 41.54 - 67.48 =	1764 ÷ (20+1) + 25.64 - 63.29 =
29	42.457 + 79.020 =	745.900 + 5.634 =	6.084 + 334.5 =
30	324.5 - 5.916 =	248.52 - 3.344 =	423.878 - 7.436 =

Supplementary Material 1 Figure 2:

Question orders post-randomisation.

No.	Test 1	Test 2	Test 3
1	86 – 54 =	5567 ÷ 41 =	1097 x 1659 =
2	21967 – 38460 =	31 x 8.3 =	712 x 472 -
3	69 x 9.2 + 24 - 36.43 =	2088 ÷ (50+8) + 41.54 - 67.48 =	2673 ÷ 81 - 74 + 132 =
4	9 – 4 =	20981 + 59215 =	52 - 1.82 ÷1.3 + 14 =
5	43 + 58 =	3980 + 4716 =	70092 + 10264 =
6	734912 – 395680 =	691324 – 384605 =	633940 + 547721 =
7	71603 ÷ 104 =	43 - 3.61 ÷ 1.9 + 31 =	142.4 - 89.25 x (3.9 + 8.4) =
8	324.5 - 5.916 =	53982 – 72106 =	574 – 693 =
9	1575 x 1169 =	76961 ÷ 81 =	75 x 2.8 =
10	42.457 + 79.020	7 + 4 =	77967 ÷ 176 =
11	5.432 x 391 =	745.900 + 5.634 =	87 x 7.4 - 142.96 + 82 =
12	71448 ÷ 104 =	43 + 61.5 ÷ (9 - 1.5) – 32 =	4.278 x 493 =
13	4 + 5 x (1 - 3) =	62 +74 =	8063 + 1468 =
14	27 - 2.24 ÷ 1.6 + 12 =	509 x 673 =	6.084 + 334.5 =
15	2079 ÷ 33 - 142 + 52	248.52 - 3.344 =	94 – 74 =
16	50494 ÷ 97 =	78 x 8.9 - 97.53 + 101 =	695 + 482 =
17	24 + 80.96 ÷ (10-1.2) – 5 =	106.14 - 61.87 x (4.5 + 5.8) =	36 + 36.57 ÷ (8 - 1.1) – 11 =
18	42385 + 38709 =	8435 – 2193 =	6593 – 1827 =
19	93 x 1.8 - 125.43 + 74 =	741320 + 567390 =	45169 ÷ 113 =
20	421 x 859 =	78 – 49 =	49607 – 62318 =
21	5 + 8 =	66650 ÷ 155 =	1764 ÷ (20+1) + 25.64 - 63.29 =
22	315 + 759 =	6 – 3 =	6 + 9 =
23	125.60 - 89.52 x (3.7 + 6.3) =	1665 x 1759 =	3 + 6 x (2 - 4) =
24	5828 ÷ 31 =	7980 ÷ 84 - 127 + 86 =	8 – 4 =
25	5731 + 4256 =	75510 ÷ 86 =	51 x 6.7 + 53 - 78.52 =
26	8546 – 2750 =	6.429 x 327 =	5678 ÷ 34 =
27	456 – 857 =	94 x 6.6 + 53 - 85.23 =	590142 – 287163 =
28	42 x 6.8 =	3 + 5 x (1 - 4) =	70835 ÷ 121 =
29	496230 + 540923 =	486 + 702 =	423.878 - 7.436 =
30	1428 ÷ (30+4) + 24.84 - 43.21 =	361 – 902 =	96 + 35 =
