# Supplementary material for: Sharing the load: How a personally coloured calculator for grapheme-colour synaesthetes can reduce processing costs
Source: PLoS One. 2021 Sep 22;16(9):e0257713. doi: 10.1371/journal.pone.0257713 (PMC8457480; doi:10.1371/journal.pone.0257713)
Supplement: S2 Fig — (PDF) [file pone.0257713.s002.pdf]

S2 Fig:

Coloured-Calculations Debrief

(1) Please order the tests by writing Test 1, Test 2 and Test 3 in the corresponding box.

Easiest				Hardest

(2) What is your gender?

Male ☐ Female ☐ Other ☐

(3) What is your age in months and years?

____

(4) What is your ethnicity?

____

(5) How many languages do you speak?

____

(6) What is the highest level of education you have?

Some high school ☐
High school ☐
Diploma or vocational training ☐
Enrolled in university as an undergraduate ☐
Complete Bachelor's degree ☐
Enrolled in a Postgraduate degree ☐
Complete a Postgraduate degree ☐
Other:_______________________________

(7) What is your highest level of mathematical education?

____

Place a cross (x) on the following scales.

(8) How much do you like mathematics?

	1	2	3	4	5	6	7	
Not at all, I hate it								Completely, I love it.

(9) How would you rate your mathematical ability?

	1	2	3	4	5	6	7	
Extremely weak.								Extremely strong.

(10) If possible, would you use a calculator that showed your colours in the future?

	1	2	3	4	5	6	7	
Never								Definitely

(11) Would you be happy to be contacted about relevant future studies, work or news in this field?

Yes ☐ No ☐

(12) If you have any questions or comments about the calculator, test or experience please leave them below with your email. The researchers will endeavour to answer them promptly.

____
____
____
____
____
____
____
____
____
____
____
____
____
____
____
____

Supplementary Figure 2:

Coloured-Calculations Debrief

(1) Please order the tests by writing Test 1, Test 2 and Test 3 in the corresponding box.

Easiest				Hardest

(2) How much do you like mathematics?

	1	2	3	4	5	6	7	
Not at all, I hate it								Completely, I love it.

(3) How would you rate your mathematical ability?

	1	2	3	4	5	6	7	
Extremely weak.								Extremely strong.

(4) If possible, would you use a calculator that showed your synesthetic colours?

	1	2	3	4	5	6	7	
No, never								Definitely

(5) Do you think other devices, that work by the same principle i.e. showing your synesthetic colours, would be useful?

	1	2	3	4	5	6	7	
No, never								Definitely

(6) Do you think, growing up, having a tool like this may have helped?

	1	2	3	4	5	6	7	
No, never								Definitely

Optional comment

____
____
____
____
____
____
____
____

(7) Would you be happy to be contacted about relevant future studies, work or news in this field?

Yes ☐ No ☐

(8) Please give a short description of the following numbers:

1	
2	
4	
12	
14	
24	
124	
142	
