# Supplementary material for: Sharing the load: How a personally coloured calculator for grapheme-colour synaesthetes can reduce processing costs
Source: PLoS One. 2021 Sep 22;16(9):e0257713. doi: 10.1371/journal.pone.0257713 (PMC8457480; doi:10.1371/journal.pone.0257713)
Supplement: S3 Fig — (PDF) [file pone.0257713.s003.pdf]

S3 Fig:

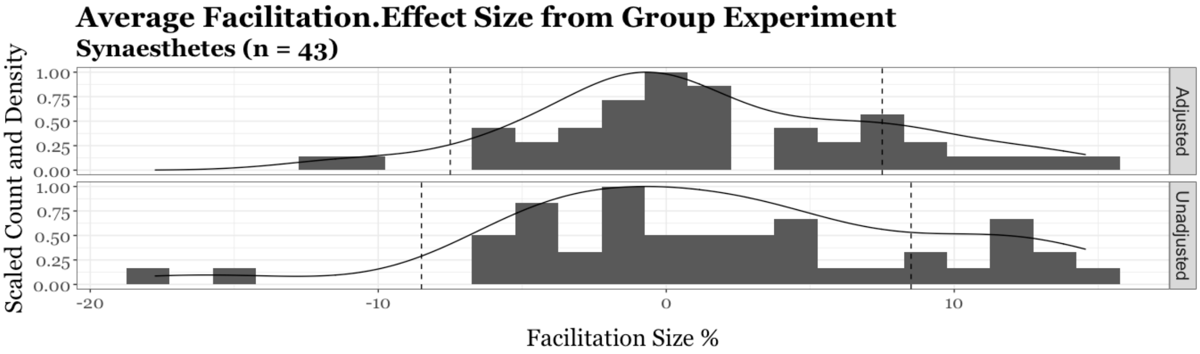

Supplementary Figure 3: Scaled Histogram and Kernel Density estimates of Facilitation Effect sizes (adjusted and unadjusted) for synaesthetes (n = 43) from the Group Experiment. The columns outside the dotted lines represent the top quartile (by magnitude) of participants.
