# Supplementary material for: Sharing the load: How a personally coloured calculator for grapheme-colour synaesthetes can reduce processing costs
Source: PLoS One. 2021 Sep 22;16(9):e0257713. doi: 10.1371/journal.pone.0257713 (PMC8457480; doi:10.1371/journal.pone.0257713)
Supplement: S1 Table — (PDF) [file pone.0257713.s004.pdf]

S1 Table

Supplementary Table 1: Group Experiment: part 1 – The Synaesthetes' (n = 53; 39 female and 14 male) descriptive statistics.

**Category:**	*Age (Years)*	*# Types of synaesthesia declared*	*Consistency Score*	*VVIQ2 Score N = 52*	*Projector /Associator score*	*Education Level*	*Mathematics Level*	*Mathematics Ability*	*Mathematics Affinity*
mean	22	5	0.7	4	-2	N/A	N/A	N/A	N/A
sd	9.2	3.9	0.23	0.7	1.4	N/A	N/A	N/A	N/A
**Med.**	21.5	5	0.6	3.9	-1.7	2	1	4	4
**Q1**	18.8	3	0.5	3.3	-2.5	2	1	3	2
**Q3**	27.7	8	0.8	4.4	-0.7	3	2	5	6

Note: a negative projector/associator score indicates an associator-type synaesthesia. Education Levels were scored thus: High School incomplete = 0, High School or equivalent diploma complete = 1, Enrolled in a Bachelor Level course = 2, Completed a Bachelor Level course = 3, Enrolled in a postgraduate degree = 4, Completed a postgraduate degree = 5. Mathematics Levels were scored thus ~ No mathematics for the final years of school = 0, Basic mathematics for the final year of school = 1, Bachelor level mathematics or advanced school mathematics = 2, Advanced university mathematics = 3. Mathematics Ability and Mathematics Affinity were self-reported on Likert scales (1 = Extremely Weak to 7 = Extremely Strong) and (1 = I hate it to 7 = I love it), respectively. Med. = median; Q1 = the first quartile; Q3 = the third quartile; and N/A = Not Applicable.

Supplementary Table 2: Group Experiment: part 2 – Synaesthetes (n = 43; 32 female and 11 male) and Non-Synaesthetes (n = 35; 32 female and 3 male) descriptive statistics.

**Category:**		*Age (Years)*	*Education Level*	*Mathematics Level*	*Mathematics Ability*	*Mathematics Affinity*
Synaesthetes	***mean***	25.77	N/A	N/A	N/A	N/A
	sd	9.9	N/A	N/A	N/A	N/A
	**Med.**	22	2	1	4	4
	**Q1**	19	2	1	3	2
	**Q3**	26	3	2	5	6
Non-Synaesthetes	***mean***	19.35	N/A	N/A	N/A	N/A
	sd	0.9	N/A	N/A	N/A	N/A
	**Med.**	19	1	2	5	5
	**Q1**	19	1	1	4	4
	**Q3**	20	1	2	6	6
