# Supplementary material for: Sharing the load: How a personally coloured calculator for grapheme-colour synaesthetes can reduce processing costs
Source: PLoS One. 2021 Sep 22;16(9):e0257713. doi: 10.1371/journal.pone.0257713 (PMC8457480; doi:10.1371/journal.pone.0257713)
Supplement: S2 Table — (PDF) [file pone.0257713.s005.pdf]

S2 Table: parameter and modelling information for the regressions.

Group experiment Part A

Model 1

Formula: Median Item Time ~ Condition + (1|ID)

Model Family: Shifted_lognormal()

Links: mu = identity; sigma = identity; ndt = identity

Priors: default brms priors

y	
nt_t (3, 2, 10)	ept
rm (0, min_Y)	decision-time-like parameter) 'ndt'
nt_t (3, 0, 10)	
nt_t (3, 0, 10)	

Data: Regression.Table (Number of observations: 159) [note: available on request]

Summary:

Group-Level Effects

~ ID (Number of levels: 53)

Estimate	Est.Error	l-95% CI	u-95% CI	Rhat	Bulk_ESS	Tail_ESS
0.35	0.07	0.23	0.50	1.01	438	871

Population-Level Effects

Parameter	Estimate	Est.Error	l-95% CI	u-95% CI	Rhat	Bulk_ESS	Tail_ESS
Intercept	1.85	0.18	1.53	2.22	1.01	446	1119
ncongruent	0.12	0.03	0.07	0.18	1.00	1166	2415
Control	0.03	0.02	- 0.02	0.08	1.00	3118	2470

Family Specific Parameters

Parameter	Estimate	Est.Error	l-95% CI	u-95% CI	Rhat	Bulk_ESS	Tail_ESS
sigma	0.12	0.02	0.08	0.17	1.00	723	1255
ndt	4.57	1.08	1.89	6.17	1.01	643	1133

Model 2

Formula: Effect Size ~ Effect Type + (1 + 1|ID)

Model Family: Skew_normal()

Links: mu = identity; sigma = identity; alpha = identity

Priors: default brms priors

y	
al (0, 4)	[illegible]
nt_t (3, 5, 11)	ept
nt_t (3, 0, 11)	
nt_t (3, 0, 11)	[illegible]

Data: Effect.Table (Number of observations: 159) [note: available on request]

Summary:

Group-Level Effects

~ ID (Number of levels: 53)

Estimate	Est.Error	l-95% CI	u-95% CI	Rhat	Bulk_ESS	Tail_ESS
5.44	1.10	3.31	7.61	1.00	1022	1316

Population-Level Effects

Parameter	Estimate	Est.Error	l-95% CI	u-95% CI	Rhat	Bulk_ESS	Tail_ESS
Intercept	7.54	1.43	4.67	10.29	1.00	2800	3697
Facilitation	- 5.60	1.69	- 8.88	2.32	1.00	3258	2695
Interference	-1.86	1.69	- 5.10	1.43	1.00	3600	2884

Family Specific Parameters

'arameter	Estimate	Est.Error	l-95% CI	u-95% CI	Rhat	3ulk_ESS	Tail_ESS
sigma	8.56	0.62	7.46	9.88	1.00	2326	2714
alpha	- 0.16	2.03	- 4.36	3.83	1.00	982	1174

Model 3

Formula: Median Item Time ~ Condition + Condition*Group + (1 | ID/Group)

Model Family: Shifted_lognormal()

Links: mu = identity; sigma = identity; ndt = identity

Priors: default brms priors

y	
nt_t (3, 2, 10)	ept
rm (0, min_Y)	decision-time-like parameter) 'ndt'
nt_t (3, 0, 10)	
nt_t (3, 0, 10)	

Data: Regression.Table.2 (Number of observations: 234) [note: available on request]

Summary:

<u>Group-Level Effects</u>

~ ID (Number of levels: 78)

Estimate	Est.Error	l-95% CI	u-95% CI	Rhat	3ulk_ESS	Tail_ESS
0.17	0.08	0.01	0.31	1.01	402	1606

~ ID:Group (Number of levels: 78)

Estimate	Est.Error	l-95% CI	u-95% CI	Rhat	3ulk_ESS	Tail_ESS
0.15	0.08	0.01	0.30	1.01	388	2058

<u>Population-Level Effects</u>

Parameter	Estimate	Est.Error	l-95% CI	u-95% CI	Rhat	Bulk_ESS	Tail_ESS
Intercept	1.88	0.14	1.62	2.19	1.00	5810	4272
ncongruent	0.00	0.02	- 0.05	0.04	1.00	10910	7909
Control	- 0.02	0.02	- 0.07	0.02	1.00	10040	7864
Congruent ynaesthetes	0.10	0.06	- 0.03	0.23	1.00	9217	7928
ncongruent ynaesthetes	0.08	0.03	0.02	0.14	1.00	8917	7514
Control ynaesthetes	0.03	0.03	- 0.03	0.09	1.00	10255	8285

<u>Family Specific Parameters</u>

Parameter	Estimate	Est.Error	l-95% CI	u-95% CI	Rhat	Bulk_ESS	Tail_ESS
sigma	0.09	0.01	0.07	0.12	1.00	5090	4291
ndt	3.41	0.90	1.26	4.79	1.00	5716	3924

Model 4

Formula: Effect Size ~ Effect Type +Effect Type*Group + (1 | ID/Group)

Model Family: Skew_normal()

Links: mu = identity; sigma = identity; alpha = identity

Priors: default brms priors

y	
al (0, 4)	
nt_t (3, 5, 11)	ept
nt_t (3, 0, 11)	
nt_t (3, 0, 11)	

Data: Effect.Table2 (Number of observations: 234) [note: available on request]

Summary:

<u>Group-Level Effects</u>

~ ID (Number of levels: 78)

Estimate	Est.Error	l-95% CI	u-95% CI	Rhat	Bulk_ESS	Tail_ESS
2.70	1.44	0.14	5.24	1.00	931	2984

~ ID:Group (Number of levels: 78)

Estimate	Est.Error	l-95% CI	u-95% CI	Rhat	Bulk_ESS	Tail_ESS
2.70	1.44	0.16	5.23	1.00	845	2263

Population-Level Effects

Parameter	Estimate	Est.Error	l-95% CI	u-95% CI	Rhat	Bulk_ESS	Tail_ESS
Intercept	- 0.20	1.48	- 3.11	2.70	1.00	7987	7460
acilitation	- 1.21	1.82	- 4.86	2.28	1.00	8661	8166
nterference	1.30	1.81	- 2.25	4.84	1.00	9151	8225
ongruency ynaesthetes	5.18	1.95	1.32	9.08	1.00	8163	8106
acilitation ynaesthetes	- 3.63	2.41	- 8.02	1.39	1.00	8737	7899
nterference ynaesthetes	- 1.89	2.43	- 6.60	2.84	1.00	9159	8367

Family Specific Parameters

Parameter	Estimate	Est.Error	l-95% CI	u-95% CI	Rhat	Bulk_ESS	Tail_ESS
sigma	7.70	0.45	6.89	8.66	1.00	6566	7235
alpha	0.69	1.15	- 1.45	2.81	1.00	6017	5651

Model 5

Formula: Effect Size ~ Effect Type + (1 | Test Number/ID)

Model Family: Gaussian()

Links: mu = identity; sigma = identity

Priors: default brms priors + to improve convergence and guard against overfitting a mildly informative normal prior (μ = 5, σ = 10), derived from the Group experiment, was specified for the intercept.

y	
al (5, 10)	ept
nt_t (3, 0, 10)	
nt_t (3, 0, 10)	

Data: Effect.Table3 (Number of observations: 126) [note: available on request]

Summary:

Group-Level Effects

~ Test Number (Number of levels: 6)

Estimate	Est.Error	l-95% CI	u-95% CI	Rhat	Bulk_ESS	Tail_ESS
3.34	2.15	0.22	8.51	1.00	789	942

~ Test Number: ID (Number of levels: 42)

Estimate	Est.Error	l-95% CI	u-95% CI	Rhat	Bulk_ESS	Tail_ESS
5.37	0.99	3.57	7.44	1.00	1413	2093

Population-Level Effects

Parameter	Estimate	Est.Error	l-95% CI	u-95% CI	Rhat	Bulk_ESS	Tail_ESS
Intercept	8.35	1.95	4.40	12.17	1.00	2054	1871
nterference	- 6.17	1.43	- 9.01	- 3.35	1.00	4659	2946
ongruency	2.26	1.14	-0.52	5.14	1.00	4812	2822

Family Specific Parameters

Parameter	Estimate	Est.Error	l-95% CI	u-95% CI	Rhat	Bulk_ESS	Tail_ESS
sigma	6.46	0.52	5.52	7.47	1.00	2788	2416
