# Supplementary material for: Sharing the load: How a personally coloured calculator for grapheme-colour synaesthetes can reduce processing costs
Source: PLoS One. 2021 Sep 22;16(9):e0257713. doi: 10.1371/journal.pone.0257713 (PMC8457480; doi:10.1371/journal.pone.0257713)
Supplement: Attachment — Submitted filename: Response.to.Reviewers.Sharing.The.Load.docx [file pone.0257713.s006.docx]

Our responses to the reviewers’ comments and questions are written in red and have been placed trailing the comments or questions, which we have reprinted in **bold**.

–––––––––––––––––––––––––––––––––––––––––––––––––––––––––––––––––––––––––––

**Reviewer 1** – **Comments** and authors’ responses

**We thank Professor Ramachandra for his thoughtful comments and recommendations.**

**The study explored the utility of a digital-color calculator (DCC) for people with grapheme-color synesthesia who sometimes may experience problems when the colors of numbers do not match their concurrent colors. The quantitative results showed an increase in calculation speed in the congruent when compared to the incongruent condition. The qualitative results were much more pronounced in that, most of the synesthetes experienced a decreased cognitive load (a sense of ease) in the congruent condition. Such quantitative and quantitative differences were not seen in the non-synesthete group. Although there has been a significant increase in the number of studies related to synesthesia, this is the first one to investigate the benefits of using a DCC. This is a well-done study and the DCC could be especially useful to many synesthetes who could be having trouble and some amount of frustration dealing with numbers in their everyday lives. This could also be beneficial to synesthetes with learning disabilities although its incidence in this population is unknown.**

**I understand that the authors were motivated by the case study SP who had a life changing experience with the introduction of DCC. However, the paper should include investigations where they have looked at the cognitive benefits of synesthesia (especially GC synesthesia).** In our Introduction we now directly point readers to a thorough review article (Ward 2013) of synaesthesia which covers such theories: “For a more detailed overview of synaesthesia in general, we suggest readers refer to *Synesthesia* in Annual Review of Psychology [8].” In our Discussion we note the body of literature describing synaesthesia’s various effects (including the cognitive effects that the reviewer describes). For example, at the individual level, ‘…some people with synaesthesia display prodigious memory as Luria noted in *The mind of a mnemonist*. Others … feel as if “…every one of [their] senses is being battered”. We note that positive and negative effects of synaesthesia have been recorded at the population level, and have referenced the literature that describes this [21,39,40]. **It is unsettling that such studies and theories such as the Dual-Coding theory have not been described in the introduction or the discussion sections.** **Some of these studies have shown only local effects (numbers, letters) but some others have shown more global effects (better vocabulary, creativity etc.).** We reference studies in our Discussion that explain the local and global benefits of synaesthesia with evidence for the *enhanced processing hypothesis*: “Indeed, as already noted, this initial cost can pay dividends in terms of memory and increased perceptual acuity by way of enhanced processing [43, 44]” **Is it possible that such cognitive advantages could be hindered by providing the non-learning-disabled synesthetes with such tools?** Yes, this is possible, but we think improbable because tools such as our DCC reinforce the perceptions of synaesthesia. Moreover, should an individual find such a tool detrimental they would be expected just to stop using it, and we do not believe there would be any permanent effects.

**In the qualitative part of the results, I see that the synesthetes have used a lot of emotive language. These results must be explained/corroborated with findings from other investigations which have described the emotional attributes of synesthesia (see Perry & Henik, 2013, and Rouw & Scholte, 2016 for example).** In our Discussion we now reference this literature. “Despite synaesthetes being known to experience increased Emotionality, they have been found not to identify, analyze, or verbalise their emotion in an increased manner [30].” In line with this, we suspect the emotive language used by the synaesthetes in this study was a response to the personal nature of the intervention rather than some general tendency to emotive language.”

**Finally, did the authors rule out cognitive, speech and language, and neurological impairments for the participants? Was any screening done (or at least asked for self-reports)?** No formal screening was done (e.g by neurological or neuropsychological examination). None of the participants presented with any gross neurological or visual impairments. However, participants were asked about their education levels and relevant affinity/ability in the field of mathematics. The participants also discussed their experiences with reading, writing, and mathematics - none declared a diagnosed deficit in these areas (in contrast to our index case). All participants had language proficiency and general cognitive ability that had enabled them to complete (or be in the process of completing) tertiary education. Moreover, none of them stood out as very extreme (cf the index case) in either the magnitude of their average response time or their response to a specific condition.

–––––––––––––––––––––––––––––––––––––––––––––––––––––––––––––––––––––––––––

**Reviewer 2** – **Comments** and authors’ responses.

**We thank Reviewer 2 for their time and effort in reviewing our paper.**

**The study investigates whether a digital calculator that represents digits in colors congruent or incongruent to the digit-color associations of grapheme-color synesthetes affects their speed of performing mathematical calculations.**

**Evaluation
I liked the idea of providing synesthetes a digital calculator that can be adjusted to represent their synesthetic color experiences. However, the study has many problems which prevent me from recommending it for publication in the present form. To address these problems would require a substantial rewrite.**

**First, on a theoretical level the claim that synesthesia “incurs an initial processing cost compared with its equivalent example from normal perception” is simply wrong. There are dozens of studies showing the automatic nature of the synesthetic experiences.** We agree that numerous studies show the automatic nature of synaesthesia. Indeed, automaticity is one of the best characteristics that distinguishes it from voluntary imagery. However, we are unsure why Reviewer 2 believes that this renders the contention about initial processing cost “simply wrong”. Many automatic processes are known to be costly; the 70 + years of experimental evidence on the Stroop Effect demonstrates how automatic processes can still incur conflict and cognitive cost.

Perhaps Reviewer 2 felt we were implying that synaesthesia is **costly overall**. We addressed this explicitly in our paper and explain our rationale: Regardless of the end effects, we assert that each synaesthetic experience bears an additional cost in the near term when compared with its equivalent example from typical perception. For example, the experience of music-colour synaesthesia *prima facie* bears some additional cost when compared with that paid for the mere ‘ordinary’ experience of hearing the music. This assertion follows from two premises. First, conscious perception is based on information processing in the brain, and this has a cost that can be framed in terms of energy. Second, synaesthesia is an unusual type of perception which has more components than normal conscious experience; namely, the concurrents.

If these premises are valid, we are of the view there should be at least some marginal processing cost to create and sustain the perceptual divergence. We do not suggest that synaesthesia is costly, *per se*. Indeed, as noted, this initial cost can pay dividends in terms of memory and increased perceptual acuity [41]. Moreover, this additional initial processing cost can be minimized at higher levels of cognition. For example, people with synaesthesia generally do not hold beliefs that their *concurrent(s)* are experienced by others; rather they often describe their *concurrent(s)* as lacking *perceptual presence* [42,43]. Irrespective of any potential benefits or cost minimizations, one defining characteristic of synaesthesia can be seen as this additional processing.

**Moreover, even the present study provides no evidence for this claim.** The aim of our study was not to provide evidence for this claim – rather, the contention follows from our stated premises (as outlined above). Our aim was to test the utility of the DCC in a group of synaesthetes. We have made the claim in framing and discussing our general explanation of how the DCC (and potential analogues) provide benefit. **If anything, the present study seems to suggest a *benefit* rather than a cost in situations in which the physical color concurs with the synesthetic experience.** We agree, this is a key conclusion, i.e. that the congruity *can* help.

**Second, the description of the method lacks important detail. For example, did the instructions emphasize speed?** Yes, we have updated our Methods description accordingly. **What exactly is represented by the dependent variable: is it the time from pressing the “next” key at the beginning of one calculation to pressing the “next” key for the next calculation?** It is the time from the beginning of the question until the ‘next’ key for the following question is pressed. We hope our updated Figure, , and its amended caption clarifies these issues. **Were the “reading”, “working”, “transcribing” phases assessed separately?** Yes, we assessed these phases separately. However, they all showed a proportional effect at the group level, with some interindividual heterogeneity. In the interests of space, we decided not to detail this sub-analysis. **The information about the sample should be presented in the paper, not in the supplementary materials.** Now included. **In both, Experiment 1 and Experiment 3 a control group of non-synaesthetes is missing (in Experiment 2 there is a control group but this group is not matched to the synaesthtete group, which makes comparisons difficult). The missing control group is particularly critical in Experiment 3. It remains completely obscure whether the improvement in task speed is specific to synesthetes or whether this might also be found in non-synesthetes.**  Reviewer 2 has raised understandable questions regarding the controls and comparisons within the experiment. First, the general design of our study can be conceptualized as one of *treatment* and *effect*, and as such calls for internal control and comparison against a *standard intervention* – i.e. the black-filled calculator in digit-colour but does not call for comparison against a second group of non-synaesthete controls. Consider the following analogy - a *treatment trial* of glucose control with a new diabetes medication in patients with diabetes compared with a current standard diabetic treatment; such a trial would only assess diabetes patients who then would form their own controls. Enrolling a group of non-diabetic patients would not help fulfill the aims of such a study.

In the second experiment we did enrol a group of non-synaesthetes as a check that observed effects – group level interference and congruency effects *–* were not merely an effect of colour (although this could still not explain the differing directions of the effect). As Reviewer 2 points out, these groups were not age and education matched, but with such direct comparison not being our goal, we believe that this control sufficed to achieve that part of our intentions.

On the same basis we did not enrol a control group for the last experiment.

**Third, for descriptive statistics it would be helpful to provide means and standard deviations in a Table.** We assume the Reviewer is referring to the Likert data that we have presented. Likert statistics represent ordinal data, and it is generally agreed that means and standard deviations do not represent appropriate summaries (although admittedly commonly encountered!). To provide an acceptable and useful summary of the central tendency of these data we reported the median in each case as (see Tables 1, 3, and our Results sections).

**Regarding statistical analysis, it is not clear for me why the results are not compared by means of repeated measures or mixed model ANOVAs. What is the benefit of the modelling approach.** In earlier analyses we performed a repeated measures ANOVA (which yielded equivalent results). However, we subsequently chose to switch to the Bayesian analysis approach for the following reason. It has the main benefit of power (particularly for the final analysis) given that the posterior distributions from the estimated regression parameters that were generated in earlier experiments can be reasonably used in the analysis of later experiments. Moreover, we chose a parameter estimation approach rather than hypothesis testing because the effects of colour in the grapheme-synaesthete population are well established and so estimating the effect sizes for the current analysis is more in line with our practical aims. **(BTW I have not found the analysis corresponding to Model 3 from S5 in the text, nor is there any justification for it?)** We agree with Reviewer 2 and have removed this exploratory model from the supplementary material.

**Notably, the values in the text and the values in Figure 3 do not correspond.** We thank Reviewer 2 for pointing this out. The model estimates for the synaesthetes’ reaction times in panel A were mistakenly superimposed on those of the non-synaesthetes. This has been rectified in the updated Figure. **Specifically, from Figure 3A it is evident that the non-synesthetes show the same performance pattern across conditions as the synesthetes (i.e., congruent < neutral < incongruent).** This resulted from the above mistake and is not reflected by the in-text data OR panel B. **This latter result makes the conclusion that synesthetes have a specific advantage from using the colored digital calculator dubious.** See above.

**Fourth, according to the PloSOne guidelines, raw data should be provided as part of the manuscript or its supporting information, or deposited to a public repository. This is not the case here.** Please find the data attached with the revised manuscript and files.
